# Women Doctors with the Army in the Field

**Published:** 1915-06

**Authors:** Helen B. Hanson


### WOMEN DOCTORS WITH THE ARMY IN THE FIELD.

BY

HELEN B. HANSON, M.D., B.S. Lond.

---

AMONGST the unusual features of the present war may perhaps be reckoned the part played by medical women in attending the wounded, and a short account of their work may prove interesting. True, the number of hospital units which they have run wholly or in part is still under twenty, and the number of women doctors concerned is probably still under fifty, yet when one considers that there are only about a thousand on the register, this is a very fair proportion.

A move was first made in this direction by Mrs. St. Clair Stobart, who during the Balkan War organised at Kirk Kilisse a hospital run entirely by women. Despite a seven days' trek in bullock wagons, the staff arrived in fit enough condition to cope with seven hundred patients in six weeks, and after the cessation of the main war the three doctors concerned stayed on as army medical officers under the Bulgarian Government.

At the beginning of the present campaign appeals were immediately made to Mrs. Stobart to organise work of a similar nature, and the Belgian authorities received with gratitude her offer to establish a unit in Brussels. University buildings were allotted as a locale, and a staff including women surgeons from London and provincial hospitals, was got together, but unfortunately Brussels was occupied by the Germans before work could be begun. After one or two threats to shoot her, Mrs. Stobart was allowed to return to England, and a second attempt to start a hospital in Antwerp under the auspices of the St. John Ambulance Association proved more successful. Here a large concert hall, situated in beautiful gardens, and accommodating one hundred and thirty patients, was provided by the Belgium Red Cross, and a party of medical women, including an X-ray expert, twelve nurses and twelve women orderlies, went out. The British Minister and the medical and military authorities all spoke equally favourably of the work done, and it must be admitted that it was accomplished under some difficulties, as after 8 p.m. only night lights and electric torches were allowed, and the wounded came in from the battlefield invariably after midnight. The theatre was the only place where black curtains permitted the use of electric light.

Before three weeks had elapsed, however, the bombardment began, and as the shells were falling all round the building, the one hundred and thirty patients had to be moved. The majority were transferred to some very inadequate cellars, and within twenty-five minutes from the beginning of operations the whole hospital (which contained thirty absolutely helpless cases) was emptied. After about twelve hours it became evident that it was impossible to continue in the cellars, both on account of their insufficient accommodation and the probable entry of the Germans, so such patients as could not stand a long journey were removed to a more commodious hospital in the city. Some who could walk betook themselves to a place of safety, and the remainder were dispatched to Ostend and England.

Belgium being now for the moment out of the question, an approach was made to the French Red Cross, who offered as a hospital the Château de Tourlaville near Cherbourg. On this occasion the unit went out with numbered Red Cross War Office brassards, under the combined auspices of the St. John Ambulance Association and the British Red Cross Society, the latter presenting the unit with an ambulance and a driver. On this occasion, too, the party took five other cars, two of them driven by women, and Dr. Florence Stoney was appointed Medical Controller. The Tourlaville offer was scarcely complied with, when a request came from the Belgian authorities to again undertake work for their wounded. It was, however, impossible to divide immediately into two, as the château accommodated some eighty patients, and the cases were very heavy. There were, however, a few Belgian wounded amongst the French, just as at Antwerp there were a few English among the Belgians. Comparing the work with Antwerp, the cases were not of course acute abdominals and chests so far from the firing-line, but owing to the closer range of the firing the wounds were far larger and more lacerated, and sepsis was extreme.

This hospital has now been running four months, and as the wounded are not now coming into Cherbourg in such great numbers, Mrs. Stobart is organising another medical women's unit for Serbia, taking with her some of the old staff. As in Antwerp, the military authorities concerned, this time Dr. Chaput from the French War Office, expressed the highest approval of the Cherbourg unit, stating in writing that the surgeons were " de meilleur valeur." The reputation of the hospital amongst the laity and the wounded is equally high, some of the old patients wishing to return from the convalescent home if only to scrub floors, and the neighbours being much impressed with the home-likeness and family spirit of the institution.

Meantime shortly after the war began Miss Garrett Anderson organised a medical women's hospital under the French Red Cross in Paris. Its locale was Claridge's Hotel, and the unit contained members of the surgical staffs of London hospitals. A great number of English officers and men as well as French occupied the one hundred and fifty beds, while the standard of the medical work can be gauged by the fact that the hospital was almost immediately recognised by the English authorities, and that subsequently Miss Garrett Anderson inaugurated a second hospital at Wimereux for seventy English soldiers directly under our War Office. The lay point of view may be gathered from the remark made by a Scotch patient, when asked if there were no men doctors, " What for would we be wanting them ? " A correspondent too, writing in the *British Medical Journal* last autumn, remarked that if British medicine were represented abroad by this one institution only, it would still be well represented ; while some humour in the situation appears when one reflects that Sir Frederick Treves announced to two women doctors last November that no English soldiers were put under the care of medical women.

Inasmuch as no prophet is without honour in his own country, the direct recognition of the Wimereux hospital by the English War Office is the greatest indication of the utility of women in war time. Kingsley's " men must work and women must weep " has long been somewhat out of date in times of peace, but many have held that as far as war went that delimitation of function still remained, at any rate for the actual area of operations. Even there, however, work of first-class importance has been found for women.

At the same time as these hospitals were at work the Scotch Federation of Woman Suffrage Societies was collecting £12,000 to equip three medical women's units for France, Belgium and Serbia respectively. The first, at Abbaye de Royaument, under Dr. Tiens, M.S., of Liverpool, has five or six doctors on the staff, amongst them an X-ray expert. It is about forty miles from the front, has now seventy patients, and is possessed of three cars, by means of which it can collect the most seriously wounded from near the field of battle. The same appreciation—medical, military and lay—has been expressed for their work as for that of the other women's hospitals.

The Belgian unit consists of three doctors and ten nurses, who have taken over the fever annexe (fifty beds) in the hospital of the Belgian Dr. Dépage in Calais. It also runs a car, and the British Red Cross gave £2,000 to the hospital. Apparently no sooner did the Belgians find Mrs. Stobart's unit unable to help than they turned to the Scotch Federation to supply medical women.

The third (Serbian) unit also consists of about six doctors, twelve nurses and twelve women orderlies. On its arrival at Kraguievatz it was presented with a hospital of two hundred and fifty wounded. It is too early yet for details of work, but more doctors, ten more nurses, and £500 more of medical stores have already been asked for and are going out.

Next, to turn to those hospitals at the front officered by men and women together.

The first of these was the British Field Hospital for Belgium (one hundred and fifty beds), which accomplished some weeks' admirable work in Antwerp before the bombardment compelled its evacuation. This hospital was remarkable for what was done in surrounding districts often under fire. For instance, on one occasion at Malines one of the women was working for seven hours with shells falling round on the roof and in the courtyard of the hospital, which was only hurriedly emptied as the Germans were entering the town.

Mention must also be made of the numerous women (medical, nursing and lay) who went out every evening in their ambulance cars to pick up the wounded. In particular a word here should be said concerning Mme. O'Gorman, who, driving her own car, brought in one hundred and twenty wounded in this way, and who escaped all dangers of shot and shell, only to succumb a month later to an illness contracted from patients in mid-France.

Other hospitals in Calais, Dunkirk, Limoges, Mentone, and several other towns in France and Serbia, have also women on their staffs, and in some cases they are in charge. As regards military hospitals at home, the Southall and Ealing Hospital has two women on its staff, and another Government hospital for infectious cases has a woman as joint pathologist. Moreover, Miss Barrie Lambert, M.D., physician in charge of the mechano-therapeutic department of the Middlesex Hospital, started with Miss French the Almeric Paget massage corps, which has supplied 115 hospitals and treated 5,000 cases (*British Medical Journal*, 30th January, 1915).

So much for medical women. But a word must be said in conclusion of those other women at the front who are caring for the sick and wounded. Their name is legion, so a brief allusion can be made to those only whose work has involved them in danger. We find, for instance, in the *British Medical Journal* of 31st October, 1914, an account of seven women in one hospital who had been hit (one fatally) while attending to the wounded in neighbouring villages. *La Française* also gives an account of a number of women, many of them sisters of mercy, who carried on their work under shell fire, and as a consequence one sister was killed and one wounded. *Jus Suffragu* also in its roll of honour makes mention of three nurses killed and two wounded, one of whom was accorded the Legion of Honour, while another nurse died from infectious disease, and more recently yet another from infectious disease in Serbia.

Lady Dorothy Fielding, who was attached to Dr. Hector Munro's "flying ambulance corps" in Belgium, and who was often under shell fire, has also with another woman been accorded the Order of Leopold.

At home, moreover, in the east coast raid the nurses in hospitals, both in Hartlepool and Scarborough, were exposed to great danger, shells falling round and in the buildings.

It is with great thankfulness that women in general, and medical women in particular, find themselves able to be of real professional value in this great crisis of their country's history.

